# Fall prediction in neurological gait disorders: differential contributions from clinical assessment, gait analysis, and daily-life mobility monitoring

**DOI:** 10.1007/s00415-021-10504-x

**Published:** 2021-03-13

**Authors:** Roman Schniepp, Anna Huppert, Julian Decker, Fabian Schenkel, Cornelia Schlick, Atal Rasoul, Marianne Dieterich, Thomas Brandt, Klaus Jahn, Max Wuehr

**Affiliations:** 1 Department of Neurology, Ludwig-Maximilians University of Munich, Marchioninistrasse 15, 81377 Munich, Germany; 2 German Center for Vertigo and Balance Disorders, Ludwig-Maximilians University of Munich, Munich, Germany; 3 Schön Klinik, Bad Aibling, Germany

**Keywords:** Neurological gait disorder, Fall risk, Gait analysis, Mobility assessment, Fall prediction

## Abstract

**Objective:**

To evaluate the predictive validity of multimodal clinical assessment outcomes and quantitative measures of in- and off-laboratory mobility for fall-risk estimation in patients with different forms of neurological gait disorders.

**Methods:**

The occurrence, severity, and consequences of falls were prospectively assessed for 6 months in 333 patients with early stage gait disorders due to vestibular, cerebellar, hypokinetic, vascular, functional, or other neurological diseases and 63 healthy controls. At inclusion, participants completed a comprehensive multimodal clinical and functional fall-risk assessment, an in-laboratory gait examination, and an inertial-sensor-based daily mobility monitoring for 14 days. Multivariate logistic regression analyses were performed to identify explanatory characteristics for predicting the (1) the fall status (non-faller vs. faller), (2) the fall frequency (occasional vs. frequent falls), and (3) the fall severity (benign vs. injurious fall) of patients.

**Results:**

40% of patients experienced one or frequent falls and 21% severe fall-related injuries during prospective fall assessment. Fall status and frequency could be reliably predicted (accuracy of 78 and 91%, respectively) primarily based on patients' retrospective fall status. Instrumented-based gait and mobility measures further improved prediction and provided independent, unique information for predicting the severity of fall-related consequences.

**Interpretation:**

Falls- and fall-related injuries are a relevant health problem already in early stage neurological gait disorders. Multivariate regression analysis encourages a stepwise approach for fall assessment in these patients: fall history taking readily informs the clinician about patients' general fall risk. In patients at risk of falling, instrument-based measures of gait and mobility provide critical information on the likelihood of severe fall-related injuries.

**Supplementary Information:**

The online version contains supplementary material available at 10.1007/s00415-021-10504-x.

## Introduction

Gait disturbances and associated falls are common complications in neurological diseases, and compromise patients' mobility, functional independence, and quality of life [1]. The prevalence for gait impairments and injurious falls is considerably higher in central compared to peripheral neurological disorders [2]. Fall-related injuries not only entail substantial medical costs but also determine patients' mortality risk [3].

Different algorithms for the assessment of fall risk in neurological gait disorders have been evaluated. Disease-specific clinical scales frequently fail to reliably predict falls in respective clinical cohorts [4, 5]. However, more general and comprehensive algorithms that include questionnaire-based surveys of sociodemographic and disease-related risk factors and clinical ratings of functional mobility (e.g., Timed-up-and-go Test) also only yield moderate validity in fall prediction [6].

Complementary approaches that focus on instrument-based measures of gait stability more reliably identify fall risk in central and peripheral neurological [7–9] and geriatric patients [10, 11]. However, quantitative gait assessment is hitherto mainly restricted to in-laboratory contexts and thus potentially underestimates challenges of daily-life mobility under which falls actually occur [12, 13]. Recent advances in real-world mobility assessment with body-worn inertial sensors therefore promise a more adequate and specific characterization of mobility impairments and identification of falls-risk-factors in neurological gait disorders [13–16].

The aim of this study was to systematically evaluate and compare the validity of different multimodal clinical assessment tools as well as quantitative measures of in- and off-laboratory mobility for the prediction of prospectively assessed occurrence, severity, and consequences of falls in 333 patients with different forms of neurological gait impairments and 63 healthy controls.

## Methods

### Participants

Patients were recruited for the cross-sectional Prospective Assessment of Falls and Mobility—study (PAss FaMous-study, DRKS-ID: DRKS00007762) in the period from June 2015 to February 2018 at the Department of Neurology and German Center for Vertigo and Balance Disorders (DSGZ) of the University Hospital Munich. Inclusion criteria were the presence of a chronic gait disorder due to (1) a peripheral vestibular disorder (i.e., chronic or subacute vestibular dysfunction according to the diagnostic criteria [17, 18]), (2) a cerebellar disorder (i.e., cerebellar ataxia according to the diagnostic criteria [19]), (3) a functional disorder (i.e., functional vertigo and dizziness or functional gait instability according to the diagnostic criteria [20, 21]), (4) a hypokinetic disorder (i.e., the diagnosis of idiopathic Parkinson´s diseases, progressive supranuclear palsy, or multiple system atrophy according to the respective diagnostic criteria [22, 23]), (5) a vascular disorder (i.e., white matter hyperintensities with cognitive and postural impairments according to the respective diagnostic criteria [24]), and (6) patients with other neurological disorders that were referred to the center due to gait instability and/or falls. The subgroup of vascular disorders mainly comprised patients with a hereditary form of microvascular encephalopathy (Cerebral Autosomal Dominant Arteriopathy with Subcortical Infarcts and Leukoencephalopathy, CADASIL) due to their mono-factorial disease etiology and their younger age that facilitate an intergroup comparison. Only patients that were able to ambulate independently were included. Independent ambulation was defined as being able to ambulate with one or two side cranes, but without dependence on a wheelchair or a wheeled walker. Only patients without any manifest motor weakness of the lower limbs (hemiparesis, paraparesis of the legs, defined by a Janda scale > 4) were included in the study. Relatives of patients and employees at the hospital were recruited as healthy controls (HC). HC were screened for confounding diseases or mobility impairments via questionnaires and via physical examination.

All participants gave their informed written consent prior to the experiments. The study protocol was approved by the local Ethics Committee (Nr. 421-13) and was conducted according to the Declaration of Helsinki.

### Clinical and fall-risk assessment at inclusion

All participants completed a standardized interview with one of the authors (R.S., J.D., C.S., and F.S.), which included a survey of the following information: ambulatory status, functional status, medication, and falls within the preceding 6 months. Medication status was categorized into ‘hypnotics’, ‘antipsychotics’, ‘antidepressants’, ‘non-opoid pain relievers’, ‘opoids’, ‘anticonvulsivants’, ‘cardiovascular drugs’, ‘cholinergic agents’, ‘anticoagulants’, and ‘hypoglycemic agents’. Retrospective fall assessment included information on: fall status (yes or no), fall frequency (no falls, occasional falls, frequent falls (> = two falls), and fall severity. A fall was defined as an unexpected event in which the person comes to rest on the ground, floor, or lower level [25]. A near fall was defined as an event that results in a marked postural instability necessitating a balance reaction or a compensating step. Falls were also graded into four different severity categories according to an established scale, i.e., the Hopkins falls grading scale (HFGS) [26]. The subjective level of stability was assessed by the Falls Efficacy Scale-International (FES-I) and the Activities-specific Balance Confidence Scale (ABC-d) [27]. Health-related quality of life was assessed by the Short Form Health Survey (SF-12) [28]. Cognitive function was screened with the Montreal Cognitive Assessment (MoCA) [29]. Each participant underwent a complete neurological and physical examination including the assessment of functional mobility by the Timed-up-Go Test (TUG) and the Functional Gait Assessment Score (FGA) [30]. All patients further completed a standardized neuro-otological testing including a comprehensive orthoptic examination, video head impulse testing, and an assessment of the subjective visual vertical. Additional diagnostic procedures [e.g., brain imaging (computer tomography and/or magnetic resonance tomography), cerebrospinal fluid diagnostics] were individually performed based on the clinical standard procedures for the underlying disease entity.

### Prospective fall assessment

Each participant was provided with falls diary covering a follow-up period of 6 months. The diary included a brief definition of different fall events and a German description of the HFGS. Participants were asked to document fall events on a daily basis (at evening) with information on: (1) the time, (2) the environmental circumstances, (3) the fall mechanisms (e.g., tripping, vertigo/dizziness, impaired consciousness, and others), (4) the duration of the post-fall lying phase, and (5) the related HFGS of each fall event [26]. Each participant was further contacted by phone on a monthly basis to cross-check and validate the documented information in the falls diary.

Based on the prospective fall assessment, participants were categorized with respect to fall status (yes or no), fall frequency [no falls, occasional falls, and frequent falls (> = two falls)], and fall severity (according to the HFGS).

### In-laboratory gait examination

In-laboratory gait assessment was performed on a 6.7-m-long pressure-sensitive carpet (GAITRite^®^, CIR System, Sparta, NJ, USA) with a sampling rate of 120 Hz. Participants walked over the carpet at their self-chosen walking speed for in total four times. Each walk was started 1.5 m in front of the mat and continued for 1.5 m beyond it to allow steady-state locomotion. Gait assessment was conducted without additional ambulatory aids. If necessary, the examiner walked beside the patient (approximately 0.3 m behind) to prevent falls. During each assessment, a sufficient number of at least 40 step events were collected.

Based on stride data recorded during the four walking trials, the following spatiotemporal gait parameters were computed: gait velocity, base of support, stride length, stride time, swing phase percentage, double support percentage, coefficient of variation (CV) of base of support, CV of stride time, CV of stride length, gait asymmetry index, and phase synchronization index [31].

### Off-laboratory mobility assessment

Following the initial visit, monitoring of daily mobility was undertaken for 14 days. Participants wore an inertial-sensor-based activity monitor (ActivPAL^®^, PAL Technologies, Glasgow), which recorded the sequence and period of time of individual bouts of ambulatory, sedentary, and sleeping behavior at a sample rate of 10 Hz. The inertial sensor was placed at the thigh of the dominant leg approximately 0.1 m cranial and 0.05 m lateral of the patella. Participants were advised to continue their daily activities as usual and not to change their routine. Upon completion of the recording period, participants removed the sensor independently and sent it back via postal service.

The following parameters (expressed as average daily estimates) were computed from the ActivPAL data in accordance to previously described procedures [32, 33] to represent characteristics of ambulatory, sedentary, and sleeping behavior: (1) daily intensity, i.e., the amount of daily energy expenditure expressed as the total metabolic equivalents (METS); (2) daily volume, i.e., the daily percentage of ambulatory, sedentary or sleeping time; (3) daily step count, i.e., the total number of steps per day; (4) daily number of sit-to-stance transitions; (5) daily pattern of ambulatory behavior computed as the exponent alpha that quantifies the distribution of bouts, with lower alpha values indicating a greater contribution of long bouts.

### Data analysis procedures

Descriptive statistics are reported as mean ± SD. In a first step, analysis of variance (ANOVA) with Bonferroni post hoc analysis and Chi square tests were used to test differences of metric and categorical parameters from clinical assessment, in-laboratory gait examination, off-laboratory mobility assessment, and retro- and prospective fall assessment between clinical groups (healthy, vestibular, cerebellar, functional, hypokinetic, and vascular). In a second step, a series of multivariate backward logistic regression analyses (controlled for disease group, age, gender, and leg length) was performed to identify independent predictors associated with three dependent fall measures of interest: (I) non-faller vs. faller, (II) occasional vs. frequent faller (defined as >  = two falls within the 6 months follow-up period), and (III) non-severe (defined as HFGS 1 or 2) vs. severe falling (defined as HFGS 3 or 4, which indicates the need for medical attention). For each of the three models, the classificatory accuracy, sensitivity, and specificity were computed. Initially, for all parameters from clinical assessment and in- and off-laboratory mobility recordings, an ANOVA was conducted for three dependent fall measures of interest. Parameters were entered into the respective regression model if the significance level of their *F* value of the ANOVA was less than 0.05. To avoid collinearity, relationships among parameters were examined using Pearson's correlations. If two parameters were strongly correlated with one another (*r* > 0.7), only the one most strongly associated with the dependent measure was retained. Further input variables into the fall regression models were the presence of previous falls (yes/no) and the ten categories of the medication status (yes/no). Statistical analysis was performed using SPSS (Version 25.0; IBM Corp., Armonk, NY).

### Data availability

Data reported in this article will be shared with any appropriately qualified investigator on request after pseudonymization.

## Results

### Demographics, clinical characteristics, and retrospective fall assessment

A total of 333 patients from six different disease groups and 63 HC were recruited. Demographics, clinical characteristics, and outcomes from retrospective fall assessment are presented in Table 1. HC and patients did not differ in age, except patients with hypokinetic disorders that were in average older (*F*_6,365_ = 2.1, *p* = 0.043). In agreement with the recruitment focus on independent ambulatory patients, functional mobility scores (i.e., FGA and TUG) of patients were in average only slightly affected, with more pronounced differences in patients with cerebellar (*p* = 0.008, *f* = 3.21, post hoc corrected) and hypokinetic disorders (*p* = 0.006, *f* = 3.84, post hoc corrected). All disease groups showed increased fear of falling (FES-I; *F*_6,365_ = 19.1, *p* < 0.001) and a lowered balance confidence (ABC-d; *F*_6,365_ = 22.2, *p* < 0.001) compared to HC. The SF-12 as marker for disease-related reduction of the quality of life was impaired in the physical function domain for all patient subgroups compared to HC (*F*_6, 365_ = 26.6, *p* < 0.001). In the psychological domain of the SF-12, only patients with hypokinetic (*p* = 0.015, *f* = 3.01, post hoc corrected) and patients with functional gait disorders (*p* = 0.013, *f* = 3.21, post hoc corrected) showed a reduction.

**Table 1:** Description of the study cohort

		Patients with									
	Healthy subjects	Vestibular disorders	Cerebellar disorders	Functional disorders	Hypokinetic disorders	Vascular disorders	Others	All	ANOVA		
Demographical characteristics									*F*(6; 365)		*p*
*n* (f/m)	63 (31/32)	88 (39/49)	93(35/58)	50 (29/21)	22 (12/10)	55 (35/20)	25 (12/13)	396	–		–
Mean age ± SD [y]	49 ± 14	58 ± 18	57 ± 18	46 ± 18	67 ± 16*	53 ± 17	51 ± 19	53 ± 18	2.1		0.043
Diagnoses		48 BVP 40 UVP	14 SCA 11 FRDA 11 EA2 18 DBN 36 SAOA 1 ARAC 1 ACM 1 post stroke	34 PPPD 16 FGD	8 IPS 4 MSA 10 PSP	51 CADASIL 4 SAE	5 BPPV 1 ED 1 EC 2 HSP 1 Liquor leakage 3 NPc 1 stroke 11 VM				
Clinical Performance Scales											
Median FGA ± SD [points]	29 ± 3	24 ± 5*	21 ± 6*	27 ± 4	17 ± 4*	26 ± 4	26 ± 4	25 ± 5	23.9		< 0.001
Mean TUG ± SD [sec]	8.8 ± 3.2	9.1 ± 2.3	11.2 ± 7.1	9.0 ± 2.0	13.7 ± 7.5	9.3 ± 2.6	9.9 ± 3.5	9.9 ± 6.5	2.0		n.s
Median MOCA ± SD [points]	29 ± 3	26 ± 4	24 ± 6*	26 ± 3	24 ± 8*	24 ± 4*	29 ± 15	26 ± 9	2.9		0.008
Subjective symptom scales											
Mean ABC-d ± SD [%]	95 ± 9	70 ± 23*	63 ± 22*	79 ± 21*	48 ± 28*	84 ± 21*	81 ± 25*	76 ± 24	22.2		< 0.001
Median FES-I ± SD [points]	17 ± 2	24 ± 9*	31 ± 13*	25 ± 8*	42 ± 19*	21 ± 7*	31 ± 13*	26 ± 12	19.1		< 0.001
Mean of SF-12 ± SD [points] physical domain	54 ± 6	40 ± 9*	38 ± 8*	38 ± 10*	33 ± 11*	47 ± 10*	40 ± 11*	43 ± 11	26.6		< 0.001
Mean of SF-12 ± SD [points] psychological domain	52 ± 9	48 ± 12	48 ± 11	44 ± 12*	38 ± 13*	47 ± 12	47 ± 10	47 ± 11	4.5		< 0.001
Retrospective falls epidemiology									df	$\chi^{2}$	*p*
No falls [%]	90	58	34	81	14	74	46	60	12	104.5	< 0.001
Occasional fall [%]	7	23	23	15	9	11	27	17			
Frequent falls [%]	3	18	43	4	76	15	27	23			
Retrospective falls severity											
Hopkins grade I [%]	45	47	24	58	10	34	29	36	24	154.5	< 0.001
II [%]	27	27	49	29	35	53	58	40			
III [%]	27	13	15	11	20	0	0	12			
IV [%]	0	12	12	0	35	11	11	12			

Sociodemographic, clinical information, and retrospective fall events (within 6 months) of the enrolled participants. Inter-subgroup differences are analyzed by ANOVA models with Sheffé post hoc comparisons (for sociodemographic and clinical data) and by $\chi^{2}$ procedures (fall data)

*Significant difference in the Sheffé post hoc comparison (compared to healthy subjects)

*f* female, *m* male, *FGA* functional gait assessment, *FES-I* falls efficacy scale-international, *TUG* timed-up-and-go test, *MOCA* Montreal Cognitive Assessment, *SF-12* short form 12, *BVP* bilateral vestibular failure, *UVP* unilateral vestibular failure, *SCA* spinocerebellar ataxia, *FRDA* Friedreich ataxia, *EA2* episodic ataxia type 2, *DBN* downbeat nystagmus syndrome, *SAOA* sporadic adult onset ataxia, *ARAC* autosomal recessive cerebellar ataxia, *ACM* tumor cerebellar, *PPPD* Persistent Phobic Postural Dizziness, *FGD* anxious functional gait disorder, *CADASIL* Cerebral Autosomal Dominant Arteriopathy with Subcortical Infarcts and Leukoencephalopathy, *SAE* subcortical arteriosclerotic encephalopathy, *BPPV* benign paroxysmal positional vertigo, *ED* encephalomyelitis disseminate, *HSP* hereditary spastic paraplegia, *NPc* Niemann Pick Disease Type c, *VM* vestibular migraine

Retrospective fall assessment revealed disease-specific differences of patients' fall status. Patients with hypokinetic and cerebellar disorders most often reported frequent falls within the last 6 months, followed by patients with vestibular and vascular disorders. Patients with functional disorders reported equally seldom frequent falling than HC but considerably more often occasional falling ($\chi^{2}$ (12) = 104.5, *p* < 0.001). Most severe consequences of falling were reported in patients with hypokinetic disorders, followed by cerebellar and vestibular disorders. In contrast, fall-related events in patients with functional disorders were most often characterized as 'near-falls' ($\chi^{2}$ (24) = 154.5, *p* < 0.001).

### In- and off-laboratory mobility assessment

Descriptive statistics of in- and off-laboratory mobility assessment can be found in Tables 2 and 3, respectively. In-laboratory gait assessment revealed general alterations of gait performance in patients, in particular a decelerated, broad-base walking pattern with increased spatiotemporal gait variability and asymmetry in comparison to HC (*p* < 0.001). Gait alterations were most pronounced in patients with hypokinetic and cerebellar disorders (*p* = 0.003, post hoc corrected). Off-laboratory monitoring of daily-life mobility revealed a general reduction of daily energy expenditure (*F*_6,376_ = 10.2, *p* < 0.001), ambulatory bout number (*F*_6,376_ = 8.6, *p* < 0.001), and step count (*F*_6,376_ = 9.5, *p* < 0.001) in patients compared to HC. The pattern of ambulatory activity was generally less variable and heterogeneous in patients (*F*_6,376_ = 3.1, *p* = 0.006). Mobility data of 26 participants were not available due to technical problems of data recording or extraction: three sensors were lost during the postal return process; for eight sensors, data could not be extracted. In ten participants, a sufficient (> 50% of daytime) measurement was possible in less than 7 days (not a fully week of mobility tracking), and five datasets were withdrawn from analysis due to multiple interruptions of more than 6 h daily.

**Table 2:** Gait performance measures

		Patients with							ANOVA	
	Healthy subjects	Vestibular disorders	Cerebellar disorders	Functional disorders	Hypokinetic disorders	Vascular disorders	Others	All	*F*(6;365)	*p*
Pace domain										
Gait velocity [m/s]	1.2 ± 0.2	1.0 ± 0.2	0.9 ± 0.2*	0.8 ± 0.2*	0.7 ± 0.3*	0.9 ± 0.3*	1.0 ± 0.2	1.0 ± 0.3	15.1	< 0.001
Stride length [m]	1.3 ± 0.2	1.1 ± 0.2	1.1 ± 0.3	1.2 ± 0.2	0.8 ± 0.2*	1.1 ± 0.2	1.2 ± 0.2	1.1 ± 0.2	16.9	< 0.001
Stride time [s]	1.1 ± 0.1	1.1 ± 0.1	1.3 ± 0.1*	1.2 ± 0.2	1.3 ± 0.1	1.1 ± 0.1	1.1 ± 0.2	1.2 ± 0.2	4.8	< 0.001
Cycle domain										
Swing phase [%]	38 ± 2	37 ± 2	37 ± 2	37 ± 2	33 ± 5 *	37 ± 2	36 ± 3	36 ± 3	9.0	< 0.001
Double support phase [%]	24 ± 4	26 ± 5*	30 ± 9*	26 ± 7	35 ± 12*	26 ± 7*	26 ± 5	27 ± 8	10.1	< 0.001
Support domain										
Base of support [m]	0.08 ± 0.03	0.12 ± 0.04*	0.15 ± 0.06*	0.12 ± 0.04*	0.11 ± 0.04*	0.11 ± 0.04*	0.11 ± 0.04*	0.12 ± 0.05	17.4	< 0.001
Base of support CV [%]	24 ± 15	24 ± 13	24 ± 12	21 ± 14*	23 ± 12	20 ± 9*	21 ± 13*	22 ± 13	1.1	n.s
Variability domain										
Stride length CV [%]	2.2 ± 1.1	3.8 ± 2.5*	5.6 ± 4.6*	3.1 ± 2.9	7.5 ± 4.2*	3.3 ± 4.2*	3.0 ± 1.3*	3.9 ± 3.0	12.9	< 0.001
Stride time CV [%]	2.3 ± 1.9	3.2 ± 1.8*	5.2 ± 1.2*	3.0 ± 2.1	7.1 ± 1.7*	3.0 ± 2.3	3.2 ± 2.2	3.7 ± 1.7	10.2	< 0.001
Asymmetry domain										
Gait asymmetry index [%]	2.5 ± 1.5	4.0 ± 2.6*	6.8 ± 3.7*	4.2 ± 2.4*	10.5 ± 4.7*	4.2 ± 2.6*	4.0 ± 2.1*	4.8 ± 2.2	9.3	< 0.001
Phase synchronization [%]	4.1 ± 1.4	5.9 ± 2.9	9.9 ± 5.3*	5.5 ± 3.3*	12.0 ± 5.6*	5.3 ± 2.5*	5.3 ± 3.2	6.7 ± 5.3	8.0	< 0.001

Outcomes from the in-laboratory gait assessment for the different clinical subgroups and healthy subjects. Spatiotemporal gait parameters are arranged with respect to the gait domains pace, cycle, support, variability, and asymmetry

*Significant difference in the Sheffé post hoc comparison (compared to healthy subjects)

*CV* coefficient of variation

**Table 3:** Daily life mobility measures

		Patients with							ANOVA model	
	Healthy subjects	Vestibular disorders	Cerebellar disorders	Functional disorders	Hypokinetic disorders	Vascular disorders	Others	All	*F*(6, 365)	*p*
Adherence										
Days recorded [d]	12.3 ± 1.9	12.2 ± 2.2	12.4 ± 1.2	12.1 ± 2.6	12.4 ± 1.3	11.8 ± 2.4	12.4 ± 1.4	12.3 ± 2.0	1.1	n.s
Time sensor 'on' [%]	96 ± 12	96 ± 6	99 ± 6	95 ± 15	98 ± 4	96 ± 12	97 ± 9	97 ± 9	0.2	n.s
Volume										
Step count [#]	10,208 ± 3,299	8,316 ± 3,293*	6,305 ± 3,302*	8,251 ± 3,162*	4,949 ± 3,606*	8,696 ± 4,265*	7,545 ± 3,941*	7,981 ± 3761	9.5	< 0.001
Ambulation time [%]	8.7 ± 2.6	7.4 ± 2.6*	5.7 ± 2.5*	7.3 ± 2.4*	4.4 ± 2.9*	7.7 ± 3.3	6.7 ± 3.1	7.0 ± 2.9	10.3	< 0.001
Sedentary time [%]	27.8 ± 7.7	31.4 ± 8.9*	34.5 ± 10.2*	28.5 ± 8.2	37.6 ± 9.7*	26.6 ± 6.7	29.2 ± 7.9	30.6 ± 9.2	7.9	< 0.001
Sleep time [%]	40.8 ± 6.7	40.9 ± 8.1	42.3 ± 9.9	43.8 ± 9.9*	41.8 ± 14.8	43.6 ± 10.7*	45.2 ± 11.6*	42.3 ± 9.5	1.1	n.s
Activity										
Ambulation bouts [#]	456 ± 134	413 ± 118	338 ± 139*	418 ± 133	247 ± 130*	439 ± 165	374 ± 174	396 ± 149	8.6	< 0.001
Ambulatory bout duration [s]	17 ± 5	16 ± 4	15 ± 4	16 ± 4	15 ± 4	15 ± 4	16 ± 4	16 ± 4	1.9	n.s
Daily intensity [MET]	35 ± 1	34 ± 1	33 ± 1*	34 ± 1	33 ± 2*	34 ± 2	34 ± 2	34 ± 2	10.2	< 0.001
Pattern										
Ambulation alpha	1.42 ± 0.04	1.43 ± 0.02	1.43 ± 0.04	1.42 ± 0.03	1.43 ± 0.03	1.43 ± 0.03	1.42 ± 0.02	1.42 ± 0.03	0.8	n.s
Sit–walk transitions [#]	40 ± 16	39 ± 16	36 ± 13	38 ± 15	30 ± 12	38 ± 17	41 ± 23	38 ± 16	1.4	n.s

Results from the 14 day continuous mobility tracking in the different clinical subgroups and healthy subjects. Mobility parameters are arranged with respect to the physical activity domains volume, activity, and pattern

*Significant difference in the Sheffé post hoc comparison (compared to healthy subjects)

### Prospective fall assessment

Falls diary information from 16% of participants was considered invalid and excluded from further analysis, due to either missing telephone contact or considerable discrepancies between falls information from the diary and monthly phone interviews. Descriptive statistics of prospective falls data from the remaining participants is presented in Table 4. Compared to HC, the percentage of fallers during follow-up was increased in all disease groups except functional and vascular disorders. Frequent falling was most often observed in patients with hypokinetic and cerebellar disorders ($\chi^{2}$ (12) = 81.4, *p* < 0.001). Most severe consequences of falling were found in patients with hypokinetic disorders ($\chi^{2}$ (24) = 38.9, *p* < 0.001), whereas the majority of fall events in functional disorders was categorized as 'near-falls'.

**Table 4:** Prospective fall assessment

		Patients with							$\chi^{2}$
	Healthy subjects	Vestibular disorders	Cerebellar disorders	Functional disorders	Hypokinetic disorders	Vascular disorders	Others	All	
*n*	56	69	80	35	19	53	20	332	
Falls epidemiology									
No falls [%]	84	64	36	86	21	79	65	66	< 0.001
Occasional fall [%]	16	20	23	9	15	11	15	17	
Frequent falls [%]	0	16	41	6	64	9	20	20	
Falls severity									
Hopkins grade I [%]	33	34	14	50	0	26	18	23	< 0.001
II [%]	33	59	63	38	50	71	73	60	
III [%]	33	2	18	12	31	3	9	12	
IV [%]	0	4	5	0	19	0	11	4	

Fall status, frequency, and severity for the different clinical subgroups and healthy subjects based on the results from the 6 month prospective fall assessment

Direct comparisons of retrospective and prospective fall data revealed occasional shifts in patients' fall status. In patients that were classified as non-fallers from retrospective assessment, 11.2% experienced occasional and 5.9% frequent falls during follow-up assessment. In patients originally classified as occasional fallers, 53.6% did not fall and 17.8% fell frequently during follow-up assessment. Finally, in patients classified as frequent fallers during retrospective assessment, 25.7% did not fall and 21.6% only occasionally fell during the follow-up period.

### Multivariate classification models for fall status, frequency, and severity

Characteristics and between-group differences of patients with respect to the categories fall status, fall frequency, and fall severity from prospective fall assessment are presented in supplementary Tables A–C.

The predictive model for fall status (non-faller vs. faller) was obtained after 9 iteration steps and yielded a correct prediction of 78% (sensitivity 62%, specificity 90%). The final model included four predictive parameters from sociodemographic and in-laboratory mobility assessment, with the most important being retrospective fall status and temporal gait variability (Table 5a). Accordingly, a positive history of falls and impaired dynamic walking stability were found to be the most important risk factors for experiencing falls during follow-up assessment.

**Table 5:** Multivariate logistic regression models for fall status, frequency, and severity

	Model information	Parameter information						
	Correct prediction	Coefficient	SE	Wald	*p* value	Exp(b)	Low	High
**A** Model I ‚fall status‘	0.78							
Retrospective fall status		1.34	0.26	26.1	< 0.001	3.45	2.28	6.34
CV of base of support		0.06	0.02	10.8	0.001	1.06	1.03	1.10
CV of stride time		0.53	0.16	5.99	0.001	1.71	1.25	2.32
Phase synchronization index		− 0,22	0.09	6.00	0.014	0.802	0.67	0.96
**B** Model II ‚frequent falls‘	0.92							
Retrospective fall status		1.36	0.33	17.2	< 0.001	3.91	2.27	8.49
MOCA		0.17	0.08	5.0	0.025	1.19	1.02	1.38
ABC-d		− 0.02	0.01	5.3	0.021	0.98	0.96	1.00
CV of stride time		0.51	0.18	8.3	0.004	1.66	1.18	2.34
Phase synchronization index		− 0.25	0.10	5.8	0.016	0.78	0.64	0.96
Ambulatory bout #		− 0.01	0.00	4.0	0.046	0.99	0.99	1.00
Daily intensity		0.42	0.21	3.9	0.047	1.52	1.01	2.30
Medication: non-opioid pain reliever		0.54	0.22	4.3	0.038	1.55	1.39	2.22
Medication: anticoagulant		0.43	0.21	3.8	0.47	1.26	1.07	1.98
**C** Model III ‚severe falls‘	0.91							
Gait velocity		− 0.07	0.03	8.4	0.004	0.93	0.89	0.98
Ambulatory bout alpha		− 33.6	8.4	6.4	0.010	0.10	0.01	0.11

Outcomes of the three multivariate logistic regression models for the categories ‘fall status’ (no falls vs. falls), ‘fall frequency’ (occasional vs. frequent falling), and ‘fall severity' (Hopkins grades I&II vs. III&IV). Regression analyses was performed on patient data only. Only parameters that significantly contributed to the model output are displayed

*FGA* functional gait assessment, *MOCA* Montreal cognitive assessment, *SF-12* short form 12, *CV* coefficient of variation, *SE* standard error

The predictive model for fall frequency (occasional vs. frequent faller) was obtained after seven iteration steps and achieved a correct prediction of 92% (sensitivity 86%, specificity 95%). The final model comprised nine predictive variables from sociodemographic, clinical, and in- and off-laboratory mobility assessment with the most influential being retrospective fall status, temporal gait variability, and intensity of daily activity (Table 5b). Thus, besides a positive history of falls and impaired dynamic walking stability, high daily activity levels were found to be an independent risk factor for frequent falling during follow-up assessment. Furthermore, the model on fall frequency was the only that comprised information on the medication status of patients (in particular pain relievers and anticoagulants) as independent risk factors.

The predictive model for fall severity (falls that do vs do not necessitate medical attention) was obtained after ten iteration steps and yielded a correct prediction of 91% (sensitivity 71%, specificity 94%). This model also considered near falls events corresponding to HFGS 1. The final model only considered two parameters from in- and off-laboratory mobility assessment, namely a reduced gait velocity and a decreased ambulatory bout alpha (Table 5c).

## Discussion

We performed a clinical and instrument-based screening of health and mobility status and prospectively monitored the occurrence and consequences of falling in a comprehensive cohort of patients with different neurological gait disorders. The main focus of this study was to identify sociodemographic, clinical, and instrument-based explanatory factors that might afford a reliable prediction of the risk of falling and fall-related consequences in this study cohort. The study focused on neurological patients with an independent ambulatory status, predominantly from a population at working age, which was reflected in only moderately impaired outcomes from clinical as well as in- and out-laboratory mobility assessment. Nonetheless, fall assessment revealed that frequent falling and severe fall-related injuries are a prevalent and relevant health problem already in the early stage of neurological gait impairments. As a direct accompanying effect of increased risk of falling, patients reported a generally lowered balance confidence and an increased risk to fall.

In agreement with previous retrospective reports [2, 34], prospective fall assessment revealed that the risk, frequency, and consequences of falling in neurological gait disorders depend on the underlying disease entity being most pronounced in central compared to peripheral and functional etiologies. The preponderance of patients with CADASIL, a hereditary, mono-factorial entity of vascular encephalopathy that causes only moderate gait changes [35] might explain the relatively low number of frequent fallers in the subgroup of vascular disorders. Falls that resulted in severe injuries were observed in more than half of patients with hypokinetic gait disorders and in approximately 25% of patients with cerebellar or peripheral vestibular disorders. In contrast, patients with functional gait disorders had a near-to-normal risk of falling, but reported a considerably higher number of near-fall events.

To identify explanatory variables that allow to predict the occurrence, frequency, and severity in the dataset of prospectively assessed fall events, we performed multiple regression analyses considering a wide set of sociodemographic characteristics, outcomes from clinical and self-report-based assessment and parameters from in- and off-laboratory gait and mobility recordings. The classificatory model on the general fall status identified fallers vs. non-fallers with an accuracy of 78%. The model included predictive factors from sociodemographic assessment and in-laboratory gait examination without, however, any contribution from off-laboratory mobility measures. A positive retrospective fall status of patients was the single most influential predictor in accordance to fall-risk assessment guidelines from the geriatric population [36]. Gait stability measures from instrument-based, in-laboratory gait examination, and functional mobility scores improved classification of patients' fall status. In particular, an increased variability and asymmetry of walking were the second most influential predictive characteristics. Both gait characteristics are established markers for gait instability [37] and associated with an increased risk of falling in geriatric patients [38], as well as patients with Parkinson's disease [37, 39], cerebellar ataxia [8, 9], or sensory deficits [40]. Moreover, increased irregularities in the base of support were predictive for a higher risk of falling. Variations in the medio-lateral control of stepping are usually considered as a stability marker of the postural, upright alignment of humans, mainly controlled by sensory inputs [41].

The classificatory model on fall frequency identified frequent vs. occasional fallers at an accuracy of 92%. Similar to the first model on fall status, prediction of fall frequency primarily relied on information on patients' retrospective fall status and instrument-based measures of dynamic gait instability, i.e., in particular the temporal variability and asymmetry of walking. However, in contrast to the first model, the frequency of fall events during follow-up assessment was further associated with measures from off-laboratory mobility assessment. In particular, a higher intensity of daily-live activity was predictive for experiencing recurrent falls during follow-up assessment. This corresponds to the previous reports that suggested that a higher amount of physical activity, especially related to household activities, is associated to an increased risk of falling in the elderly population [42, 43]. Similar, higher levels of physical activity in patients with early Parkinson's disease were shown to be linked to an increased risk of experiencing falls during ambulation [44]. Comparison between the number of ambulatory bouts and the overall daily intensity in patients that experience frequent falls indicates that their overall ambulatory activity is constituted by fewer but considerably longer bouts of walking. Finally, the cognitive status of patients was predictive for frequent fall status. A decline in cognitive resources is usually thought to be associated increased risk of falling [45, 46]. In contrast, the current model suggests an inverse relationship in that higher cognitive resources were found to be predictive for falling during the follow-up assessment. This counterintuitive observation might be explained by the fact that cognitive decline not only impairs gait functionality per se but is also associated with a general reduction in outdoor mobility in the population [47, 48]. It is thus conceivable that moderate cognitive deficits (as observed in the present clinical cohort) might result in a less frequent exposure of patients to complex outdoors balance situations and thereby actually protect patients against falling [44]. Finally, we found that the medication status of patients (in particular non-opioid pain relievers and anticoagulants) appears to be relevant for identifying frequent fallers. However, the contribution of the medication status to fall-risk prediction in our cohort is certainly less significant as previously demonstrated for geriatric cohorts [49]. In addition, the commonly described ‘fall risk increasing drugs (FRIDs)’ such as hypnotics, antipsychotics, and antidepressants were not found to be relevant for fall-risk prediction in our cohort.

Taken together, these findings suggest that in particular patients with early stage gait impairments that yet maintain near-to-normal levels of daily activity are at a high risk of experiencing recurrent falls during ambulation. This risk will decrease not until advanced stages of disease that are linked to considerable reduced levels of mobility and daily activity [44]. However, a clinical advice to reduce ambulatory activity in early stage neurological gait disorders to protect patients from recurrent falling would not be appropriate due to the apparent neuroprotective effects of activity in these patients [50, 51]. Therefore, a balance must be found between maintaining activity and applying protective measures that specifically minimize the risk of severe fall-related injuries in these patients.

The third classificatory model on fall severity identified those patients who experienced falls with severe consequences (i.e., fall-related injuries that required medical attention) during follow-up assessment with an accuracy of 91%. Fall severity classification was only based on two outcome measures from in- and off-laboratory mobility assessment. An impaired pattern of daily ambulatory activity (i.e., ambulatory bout alpha) had the highest influence on predicting severe consequences of falling. Accordingly, patients susceptible to severe falls exhibited a lower variance of ambulatory activity with a reduced number of by tendency longer ambulatory bouts. This might involve an impaired ability to adjust ambulation activity to altered surrounding conditions. In line with this, a reduced variance in motor performance has been generally associated with age- or disease-related functional decline and maladaptive responses to changing environmental demands [52]. In addition, severe falling was associated with a lower habitual walking speed as assessed during in-laboratory gait examination, in accordance with the previous reports that suggest habitual walking speed as a functional parameter for morbidity, mortality, and social functionality [53]. Patients that experienced falling with severe injuries had significantly higher outcomes in the disease-related reductions of quality of life as assessed by the SF-12. This emphasizes that fall events and related injuries already significantly affect the quality of life in patients with moderate neurological gait disorders. Furthermore, this effect appears to be more related to the severity rather than the mere frequency of falls.

The findings of this study should be interpreted with respect to certain limitations: first, patients with hypokinetic disorders were in average older than other patients and controls, which might at least partly explain their apparently lower levels of physical activity. Second, falls, gait, and mobility impairments in middle-aged patients with vascular encephalopathy (CADASIL) in the subgroup of vascular disorders are likely less pronounced compared to advanced-aged patients with sporadic vascular encephalopathy [35]. However, recruitment of patients with sporadic vascular encephalopathy was not eligible particularly due to the common presence of a multi-factorial etiology in these patients. Finally, we could not include disease-specific scores (e.g., UPDRS and SARA) into the classificatory falls models due to mixed-disease model design. However, instead, we used the FGA—a general rating scale to disease-related gait and mobility impairments—which facilitates a consistent rating of patients across different disease groups.

Taken together, this prospective fall assessment study yielded three classificatory models that allow to predict patients' general fall status, their frequency of falling, and the severity of fall-related injuries at a high accuracy between 78 and 91%. Importantly, all three models were found to be independent of the specific disease group and thus equally apply for neurological gait disorders of central, peripheral, and functional pathogenesis. Clinical and instrument-based outcome measures had a differential impact on outcome prediction within and between the three classificatory models. These differential contributions encourage and provide guidelines for a multi-level, stepwise approach for fall-risk assessment in patients with neurological gait disorders that may help adjusting fall prevention strategies. Accordingly, basic index information that is readily available from medical history taking allows a good estimation on the general risk of falling and may promptly inform the clinician which patient would or would not benefit from a more in-depth examination. For those patients at a general risk of falling, a more elaborate, instrument-based gait and mobility examination provides additional, unique information with respect to the severity of their fall susceptibility and the likelihood for the occurrence of severe fall-related injuries.

## Supplementary Information

Below is the link to the electronic supplementary material.

Supplementary file1 (DOCX 37 KB)
