# Supplementary material for: Fall prediction in neurological gait disorders: differential contributions from clinical assessment, gait analysis, and daily-life mobility monitoring
Source: J Neurol. 2021 Mar 13;268(9):3421–34. doi: 10.1007/s00415-021-10504-x (PMC8357767; doi:10.1007/s00415-021-10504-x)
Supplement: Supplementary file 1 — Supplementary file1 (DOCX 37 KB) [file 415_2021_10504_MOESM1_ESM.docx]

Supplemental Table A ANOVA Fall Model I ‘non-faller vs. faller’ (only patient data included)

|  |  |  |  | **ANOVA** | |
| --- | --- | --- | --- | --- | --- |
| n = 279 | non-faller | faller | df | **F(1,278)** | **p** |
| **Clinical measures** |  |  |  |  |  |
| FGA [points] | 25.2 ± 4.9 | 20.8 ± 6.0 | 1 | 36.3 | <0.001 |
| TUG [sec] | 9.2 ± 2.7 | 11.5 ± 7.1 | 1 | 13.9 | <0.001 |
| MOCA [points] | 25.0 ± 2.1 | 24.9 ± 4.3 | 1 | 0.01 | n.s |
| ABC-d [%] | 78.4 ± 22.5 | 61.9 ± 24.7 | 1 | 30.1 | <0.001 |
| FES-I [points] | 24.2 ± 10.3 | 32.6 ± 14.7 | 1 | 28.4 | <0.001 |
| SF-12 [points] | 30.8 ± 3.5 | 31.4 ± 7.7 | 1 | 0.8 | n.s. |
| **Gait measures** |  |  |  |  |  |
| gait velocity [m/s] | 1.06 ± 0.25 | 0.84 ± 0.33 | 1 | 40.1 | <0.001 |
| stride length [m] | 1.18 ± 0.20 | 1.00 ± 0.29 | 1 | 34.3 | <0.001 |
| stride time [s] | 1.12 ± 0.16 | 1.31 ± 0.46 | 1 | 20.8 | <0.001 |
| swing phase [%] | 37.2 ± 2.2 | 34.2 ± 5.7 | 1 | 36.2 | <0.001 |
| double support phase [%] | 25.4 ± 4.2 | 31.6 ± 11.2 | 1 | 40.4 | <0.001 |
| base of support [m] | 0.11 ± 0.04 | 0.14 ± 0.06 | 1 | 14.0 | <0.001 |
| base of support CV [%] | 20.3 ± 10.7 | 24.6 ± 14.5 | 1 | 7.9 | 0.005 |
| stride length CV [%] | 3.2 ± 2.1 | 6.4 ± 4.4 | 1 | 46.9 | <0.001 |
| stride time CV [%] | 2.9 ± 1.9 | 6.2 ± 2.7 | 1 | 48.1 | <0.001 |
| gait asymmetry index [%] | 4.2 ± 3.8 | 7.7 ± 4.9 | 1 | 23.3 | <0.001 |
| phase synchronization [%] | 5.4 ± 3.8 | 10.5 ± 5.3 | 1 | 26.6 | <0.001 |
| **Mobility measures** |  |  |  |  |  |
| step count [#] | 8,020 ± 3,607 | 7,040 ± 3,980 | 1 | 4.0 | 0.047 |
| ambulation time [%] | 7.1 ± 2.8 | 6.2 ± 3.0 | 1 | 4.7 | 0.031 |
| sedentary time [%] | 30.6 ± 9.1 | 32.1 ± 9.8 | 1 | 1.5 | n.s. |
| sleep time [%] | 42.7 ± 9.4 | 42.4 ± 10.7 | 1 | 0.1 | n.s. |
| ambulation bouts [#] | 406 ± 142 | 352 ± 151 | 1 | 7.9 | 0.005 |
| ambulatory bout duration [s] | 15.3 ± 3.8 | 15.4 ± 4.4 | 1 | 0.1 | n.s. |
| daily intensity [MET] | 33.8 ± 1.5 | 33.4 ± 1.6 | 1 | 3.3 | n.s. |
| ambulation alpha | 1.42 ± 0.03 | 1.43 ± 0.04 | 1 | 2.8 | n.s. |
| sit-walk transitions [#] | 38.7 ± 16.2 | 36.7 ± 16.0 | 1 | 1.0 | n.s. |

Legend: ANOVA outcomes for the category ‘fall status’. Only parameters that showed a significant between-group difference with respect to this category were included in the subsequent multivariate logistic regression analysis.

Abbr: FGA – functional gait assessment, FES-I – falls efficacy scale – international, TUG – timed-up-and-go test, MOCA –Montreal Cognitive Assessment, SF-12 – short form 12, CV – coefficient of variation

Supplemental Table B ANOVA Fall Model II ‘occasional vs. frequent falls’ (only patient data included)

|  |  |  |  | **ANOVA** | |
| --- | --- | --- | --- | --- | --- |
| n = 103 | occasional | frequent | df | **F(1, 102)** | **p** |
| **Clinical measures** |  |  |  |  |  |
| FGA [points] | 24.4 ± 5.5 | 20.3 ± 5.3 | 1 | 22.5 | <0.001 |
| TUG [sec] | 9.5 ± 3.2 | 12.1 ± 8.3 | 1 | 12.5 | <0.001 |
| MOCA [points] | 25.0 ± 3.2 | 24.8 ± 10.8 | 1 | 0.1 | n.s |
| ABC-d [%] | 76.3 ± 23.8 | 57.6 ± 22.2 | 1 | 29.0 | <0.001 |
| FES-I [points] | 25.4 ± 11.8 | 34.3 ± 13.7 | 1 | 23.5 | <0.001 |
| SF-12 [points] | 30.8 ± 3.5 | 32.1 ± 9.5 | 1 | 2.5 | n.s. |
| **Gait measures** |  |  |  |  |  |
| gait velocity [m/s] | 1.02 ± 0.27 | 0.81 ± 0.34 | 1 | 25.6 | <0.001 |
| stride length [m] | 1.15 ± 0.23 | 0.97 ± 0.30 | 1 | 37.8 | <0.001 |
| stride time [s] | 1.16 ± 0.23 | 1.32 ± 0.52 | 1 | 12.9 | <0.001 |
| swing phase [%] | 36.8 ± 3.1 | 33.7 ± 6.2 | 1 | 30.8 | <0.001 |
| double support phase [%] | 26.4 ± 6.1 | 32.6 ± 11.9 | 1 | 31.9 | <0.001 |
| base of support [m] | 0.11 ± 0.04 | 0.14 ± 0.06 | 1 | 18.9 | <0.001 |
| base of support CV [%] | 21.6 ± 11.9 | 23.6 ± 14.3 | 1 | 1.4 | n.s. |
| stride length CV [%] | 3.7 ± 3.2 | 6.9 ± 4.9 | 1 | 38.8 | <0.001 |
| stride time CV [%] | 3.5 ± 2.4 | 6.6 ± 3.4 | 1 | 42.2 | <0.001 |
| gait asymmetry index [%] | 4.6 ± 3.9 | 8.5 ± 7.9 | 1 | 21.1 | <0.001 |
| phase synchronization [%] | 6.2 ± 4.9 | 11.6 ± 8.4 | 1 | 24.0 | <0.001 |
| **Mobility measures** |  |  |  |  |  |
| step count [#] | 7,680 ± 3,676 | 7,234 ± 4,035 | 1 | 1.4 | n.s. |
| ambulation time [%] | 7.0 ± 2.9 | 6.1 ± 3.1 | 1 | 4.2 | 0.039 |
| sedentary time [%] | 31.4 ± 9.5 | 30.6 ± 9.2 | 1 | 0.3 | n.s |
| sleep time [%] | 42.1 ± 9.1 | 44.2 ± 12.0 | 1 | 2.0 | n.s. |
| ambulation bouts [#] | 387 ± 143 | 356 ± 157 | 1 | 4.1 | 0.040 |
| ambulatory bout duration [s] | 15.4 ± 4.0 | 16.7 ± 4.4 | 1 | 0.4 | n.s. |
| daily intensity [MET] | 32.9 ± 1.5 | 33.7 ± 1.6 | 1 | 4.9 | 0.029 |
| ambulation alpha | 1.44 ± 0.03 | 1.41 ± 0.03 | 1 | 6.9 | 0.009 |
| sit-walk transitions [#] | 39.0 ± 16.6 | 34.7 ± 14.1 | 1 | 3.0 | n.s. |

Legend: ANOVA outcomes for the category ‘fall frequency’. Only parameters that showed a significant between-group difference with respect to this category were included in the subsequent multivariate logistic regression analysis.

Abbr: FGA – functional gait assessment, FES-I – falls efficacy scale – international, TUG – timed-up-and-go test, MOCA –Montreal Cognitive Assessment, SF-12 – short form 12, CV – coefficient of variation

Supplemental Table C ANOVA Fall Model III ‘non-severe vs. severe falling’ (only patient data included)

|  |  |  |  | **ANOVA** | |
| --- | --- | --- | --- | --- | --- |
| n = 103 | Hopkins Grade I & II | Hopkins Grade III & IV | df | **F(1,102)** | **p** |
| **Clinical measures** |  |  |  |  |  |
| FGA [points] | 22.7 ± 5.8 | 17.9 ± 75.6 | 1 | 10.6 | 0.001 |
| TUG [sec] | 10.6 ± 5.8 | 13.0 ± 7.7 | 1 | 3.2 | n.s. |
| MOCA [points] | 24.5 ± 3.9 | 26.0 ± 10.4 | 1 | 0.8 | n.s |
| ABC-d [%] | 69.4 ± 23.9 | 51.6 ± 26.4 | 1 | 11.3 | 0.001 |
| FES-I [points] | 28.2 ± 11.9 | 39.5 ± 17.4 | 1 | 15.3 | <0.001 |
| SF-12 [points] | 30.6 ± 3.5 | 33.5 ± 10.2 | 1 | 4.1 | 0.043 |
| **Gait measures** |  |  |  |  |  |
| gait velocity [m/s] | 0.98 ± 0.29 | 0.72 ± 0.32 | 1 | 19.0 | <0.001 |
| stride length [m] | 1.12 ± 0.24 | 0.90 ± 0.28 | 1 | 16.4 | <0.001 |
| stride time [s] | 1.19 ± 0.28 | 1.37 ± 0.59 | 1 | 6.5 | 0.012 |
| swing phase [%] | 36.3 ± 3.3 | 32.4 ± 6.3 | 1 | 18.8 | <0.001 |
| double support phase [%] | 27.4 ± 6.9 | 33.7 ± 12.6 | 1 | 13.5 | <0.001 |
| base of support [m] | 0.12 ± 0.05 | 0.15 ± 0.06 | 1 | 10.3 | 0.002 |
| base of support CV [%] | 23.8 ± 13.3 | 22.6 ± 15.1 | 1 | 0.2 | n.s. |
| stride length CV [%] | 4.5 ± 3.8 | 6.5 ± 3.6 | 1 | 6.2 | 0.014 |
| stride time CV [%] | 4.0 ± 3.2 | 7.1 ± 5.2 | 1 | 13.8 | <0.001 |
| gait asymmetry index [%] | 5.5 ± 4.8 | 9.2 ± 6.1 | 1 | 6.5 | 0.011 |
| phase synchronization [%] | 7.0 ± 5.6 | 12.2 ± 8.7 | 1 | 12.3 | 0.001 |
| **Mobility measures** |  |  |  |  |  |
| step count [#] | 7,891 ± 4,156 | 5,301 ± 2,972 | 1 | 8.4 | 0.004 |
| ambulation time [%] | 7.0 ± 3.2 | 4.9 ± 2.4 | 1 | 9.3 | 0.003 |
| sedentary time [%] | 30.3 ± 9.3 | 35.1 ± 10.8 | 1 | 5.0 | 0.026 |
| sleep time [%] | 43.4 ± 10.5 | 39.5 ± 10.0 | 1 | 2.9 | n.s. |
| ambulation bouts [#] | 393 ± 152 | 279 ± 128 | 1 | 11.9 | 0.001 |
| ambulatory bout duration [s] | 15.4 ± 4.3 | 15.2 ± 4.4 | 1 | 0.02 | n.s. |
| daily intensity [MET] | 33.8 ± 1.7 | 32.8 ± 1.3 | 1 | 6.6 | 0.011 |
| ambulation alpha | 1.44 ± 0.03 | 1.40 ± 0.03 | 1 | 9.2 | 0.002 |
| sit-walk transitions [#] | 37.4 ± 15.6 | 33.6 ± 13.0 | 1 | 1.2 | n.s. |

**Legend:** ANOVA outcomes for the category ‘fall severity’. Only parameters that showed a significant between-group difference with respect to this category were included in the subsequent multivariate logistic regression analysis.

Abbr: FGA – functional gait assessment, FES-I – falls efficacy scale – international, TUG – timed-up-and-go test, MOCA –Montreal Cognitive Assessment, SF-12 – short form 12, CV – coefficient of variation
